# Using transcription and translation regulators to improve recombinant protein expression in CHO cells: Optimizing CHO cell protein expression via transcription and translation regulators

**DOI:** 10.3724/abbs.2025160

**Published:** 2025-09-18

**Authors:** Shaolei Geng, Chunliu Mi, Chong Wang, Jiayue Li, Qiuli Sun, Weidong Li, Tianyun Wang

**Affiliations:** 1 International Joint Research Laboratory for Recombinant Pharmaceutical Protein Expression System of Henan Xinxiang Medical University Xinxiang 453003 China; 2 Henan Key Laboratory of Neurorestoratology and Protein Modification the First Affiliated Hospital of Henan Medical University Xinxiang 453003 China; 3 Henan Engineering Research Center for Biopharmaceutical Innovation Xinxiang Medical University Xinxiang 453003 China; 4 School of Medical Engineering Xinxiang Medical University Xinxiang 453003 China

**Keywords:** recombinant therapeutic protein, Chinese hamster ovary cell, transcription factor engineering, translational regulators, cell engineering

## Abstract

Chinese hamster ovary (CHO) cells are the predominant platform for the production of recombinant therapeutic proteins (RTPs). Over the past two decades, numerous strategies have focused on increasing the growth, titer and quality of RTPs in CHO cells and concomitantly reducing production costs. Transcription and translation are continuous processes that are crucial for RTP production. Transcription factors (TFs) are predominantly involved in the regulation of growth, metabolism, and endoplasmic reticulum function, and TF engineering has been demonstrated to be an efficacious strategy for enhancing RTP expression, enabling the design of customized TFs and promoters. Translation regulators encompass translation, folding, secretion, and post-translational modifications and involve a multitude of key genes and signaling pathways, which are vital for the immunogenicity of RTPs. Novel synthetic biology methods and advancements in genomics have played significant roles in the design of specific TFs and the selection of translation factors. This review summarizes the strategies for increasing RTP expression in CHO cells via TFs and translation factors. We also propose methods to further optimize protein expression strategies and develop more efficient CHO cell lines by leveraging advancements in genomics and synthetic biology research.

## Introduction

Biomedicine has become a fundamental component of the healthcare industry and has been a rapidly growing industry worldwide, with market growth exceeding $300 billion by 2025 [ 1, 2]. Recombinant therapeutic proteins (RTPs) are an important part of biomedicine, and the increasing demand for RTPs has prompted biopharmaceutical companies to seek ways to optimize the manufacturing process.

Currently, RTPs are produced mainly by mammalian cell expression systems [ 3, 4]. Compared with other host cells, the widely used mammalian cells include human embryonic kidney (HEK) cells, PER C6 immortalized primary human embryonic retina cells, NS0 mouse myeloma cells, baby hamster kidney (BHK) cells, SP2/0 mouse myeloma cells, and Chinese hamster ovary (CHO) cells, rendering them the ideal choice for generating RTPs, especially in clinical settings [ 5, 6]. Considering the expression levels, biological activity, safety and cost of RTPs, the mammalian cell expression system has become the best choice [7]. After decades of development, the increasing demand for RTPs has challenged the expression levels of RTPs in mammalian cells.

CHO cells have undergone continuous domestication and modification and have developed into various subtypes, including CHO-S, CHO-K1 CHO-GS and CHO-DG44. Moreover, the methotrexate selection systems which are based on dihydrofolate reductase (DHFR) [8] and the methionine sulfoxide selection systems which are based on glutamine synthetase (GS) [9] have been developed and are widely used in industrial production. The recombinant proteins produced from CHO cells have post-translational modifications (PTMs) similar to those in humans, which are important for their effectiveness and immunogenicity [ 10, 11]. In addition, CHO cells can be grown in suspension at a large scale and high density, enabling efficient replication and expression of transgenes [7]. Over 89% of newly approved recombinant antibodies are produced with CHO cells [ 12– 14]. Some monoclonal antibody (mAb) titers have exceeded 10 g/L in perfusion or fed-batch cultures of CHO cells [ 15, 16]. Compared with CHO cells, HEK293 cells possess a more refined capacity for complex PTMs, offering significant advantages when reducing immunogenicity or extending the half-life of mAbs. Consequently, HEK293 cells are employed primarily in the research and development phase of mAb production. A comparison of mAbs currently produced in CHO and HEK293 cells is presented in Table 1. However, the cost of producing RTPs in CHO cells remains relatively high, and the expression levels of some proteins, such as difficult-to-express (DTE) proteins, bispecific antibodies, and cytokines, remain low and challenging [ 17, 18]. This requires further strategies to improve yields and achieve successful expression of RTPs in CHO cells.

**Table:** **Table 1** Current therapeutic antibodies produced in CHO cells versus HEK293T cells

Cell line	mAbs	Target	Access
CHO cells	Donanemab (Kisunla®)	Amyloid β	https://pi.lilly.com/us/kisunla-uspi.pdf
	Axatilimab (Niktimvo®)	CSF-1 R	https://www.accessdata.fda.gov/drugsatfda_docs/label/2024/761411s000lbl.pdf
	Crovalimab (PiaSky®)	Complement C5	https://www.gene.com/download/pdf/piasky_prescribing.pdf
	Tarlatamab	DLL3, CD3	https://www.accessdata.fda.gov/drugsatfda_docs/label/2024/761344s000lbl.pdf#page=31
	Zanidatamab (Ziihera®)	HER2	https://pp.jazzpharma.com/pi/ziihera.en.USPI.pdf
	Zenocutuzumab	HER2, HER3	https://www.bizengri.com/pdf/pi.pdf
	Odronextamab (Ordspono®)	CD20, CD3	https://ec.europa.eu/health/documents/community-register/2024/20240822163419/anx_163419_en.pdf
	Vilobelimab	Complement C5a	https://www.fda.gov/media/166824/download?attachment
	Socazolimab	PD-L1	https://www.doc88.com/p-63447921192985.html
HEK293T	Adalimumab	TNF-α	https://pubmed.ncbi.nlm.nih.gov/30468855/
cells	Bispecific antibody iMab-N6	HIV-1	https://pubmed.ncbi.nlm.nih.gov/36071449/
	Fc-silent chimeric antibody	CD36	https://pubmed.ncbi.nlm.nih.gov/39442990/
	Anti-cholerae monoclonal antibody	Cholera	https://pmc.ncbi.nlm.nih.gov/articles/PMC11637869/

The expression level of recombinant proteins depends on both the maintenance time of viable cell density (VCD) and the maximum specific protein productivity achieved in cell culture [ 19 – 21]. To achieve these goals, strategies such as inhibiting apoptosis to extend the culture stage [22], applying environmental stress [ 23, 24], or implementing perfusion/fed-batch systems [25] have been developed to improve final yields and quality. However, some cell lines are unrelated in terms of DNA copy number and target mRNA levels, along with transcriptional instability and low efficiency [26]. Epigenetic modifications such as histone deacetylation and DNA methylation reduce gene transcription ability [27]. Additionally, excessive RTP expression decreases the ability of the endoplasmic reticulum (ER) to correctly fold heterologous proteins, resulting in aggregation and affecting protein secretion and quality. Therefore, attention should be given to the integration of transcription and translation regulation, and new strategies should be developed to further improve and control the performance of CHO cells.

Recent advances in cell biology, biochemistry, and bioengineering have revolutionized the process of producing RTPs in CHO cells. In this review, we summarize the current strategies for improving the yields and quality of RTPs in CHO cells through transcription and translation factor engineering and explore ways to refine and optimize future directions for this system.

## Transcription and Translation Regulators

Transcription and translation are the two most important processes in RTP production; these processes enable cells to express the information encoded in DNA and control protein location, conformation, and stability [28]. A powerful method for engineering CHO cells and enhancing their productive phenotype is the use of transcription factors (TFs) which activate/inhibit multiple targets and change cell phenotypes [29]. Global translational control of translation significantly determines RTP output in cells [17]. mRNA stability and translation efficiency constitute two key aspects of the posttranscriptional process that profoundly affect protein production [30]. Newly synthesized peptides are then transported to the ER, where they undergo correct folding and PTMs regulated by factors such as activating transcription factor (ATF), protein disulfide isomerase (PDI) and X-box binding protein 1 (XBP-1), affecting protein aggregation [ 24, 31]. Subsequently, glycosylation and other modifications occur in the Golgi apparatus to form functional proteins which are then secreted into the cellular supernatant.

### Screening of transcription and translation regulators

For a given DNA sequence, the potential TFs for binding can be predicted using online software, such as Jaspar 2022 [32]. With the development of biotechnology, transcriptomics, phosphorylated proteomics, chromatin immunoprecipitation technology and zinc finger protein TF (ZFP-TF) libraries have been used to analyze industrial CHO cell lines to calculate predictive TFs in combination with transcription elements and analyze upstream regulatory factors [33]. RNA-seq is commonly used for transcriptome analysis, providing detailed insights into intracellular transcription dynamics. Different mRNA expression patterns and levels often occur in cells and alter mRNA transcription stability and translation efficiency by increasing transcriptional activity. RNA metabolic sequencing using thiol (SH)-linked alkylation is applicable to diverse cell states and lines and allows the dynamics underlying TFs to be revealed by metabolic markers [34]. Nuclear proteomic and phosphoproteomic analyses play important roles in screening transcription and translation factors and regulating the final productivity of RTPs, with TF phosphorylation sites serving as potential engineering targets. In addition, the epigenome is an effective method for screening translation factors, such as N6-methyladenosine (m ^6^A), demonstrating its role in the regulation of mRNA splicing, stability, and translation. The cooperative actions of the 5-member T521-B homology (YTH) domain-containing protein reader family (YTHDF1 and YTHDF3) enhance translation, demonstrating the effectiveness of epigenetic transcriptomics for improving RTPs and viral vector production in engineered cell lines [35]. Using genome-wide CRISPR knockout sequencing, Barlan *et al*. [14] screened two well-known fucosylation regulators, α-1,6-fucosyltransferase (FUT8) and solute carrier family 35 member C1 (SLC35C1), which significantly alter the fucosylation of RTPs in CHO cells.

### Classification and function of transcription factors

TFs in CHO cells can be categorized into endogenous, heterologous and synthetic TFs on the basis of artificial intelligence and modular characteristics. Synthetic TFs, such as artificial ZFP-TFs, are engineered from combinatorial libraries and fused to effective transcriptional activators/repressors. This design enhances the specificity of transgenic promoters, providing an ideal platform for multipathway gene regulation in CHO cells. ZFP-TFs can recognize and activate promoters containing target DNA-binding domains with high specificity [36]. Construction strategies include transcription activator-like effect-TFs, CRISPR-TFs, chimeric TFs and zinc finger-ZFs. Notably, chimeric TFs leverage bacterial DNA-binding domains fused to mammalian transcriptional silencing domains, enabling highly efficient induction of gene expression systems via evolved DNA-binding responses [37].

**Figure 1:**
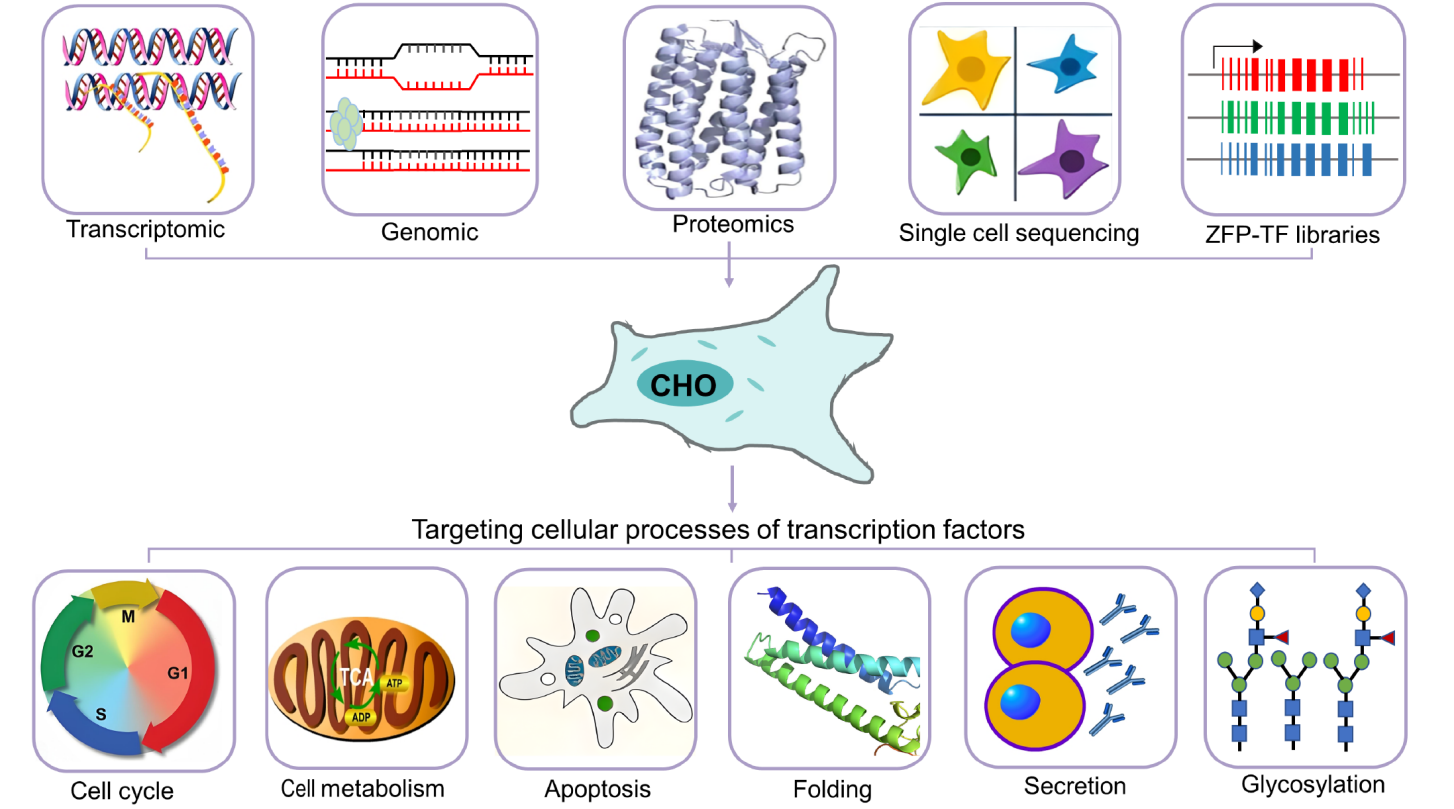
Figure 1 Transcription factor engineering in CHO cells improves RTP expression Targeted engineering regulates genes that affect protein expression processes such as the cell cycle (red: G1 phase; blue: S phase, green: G2 phase; yellow: M phase), apoptosis, the tricarboxylic acid cycle (TCA, mitochondrial green arrow), glycosylation, protein folding and secretion (blue ellipse: nucleus; yellow area: cytoplasm; purple circle: membrane; Y-shaped structure: secreted antibodies). Global screening methods for transcription factors reviewed in CHO cells are shown at the top, including genomics (dark green represents the nucleosome), transcriptomics (color-coded DNA double helix), proteomics, single-cell sequencing (differentially colored cells), and zinc finger protein (TF) libraries (red/green/blue: genomic transcriptional regions).

The classification of TFs according to their roles in regulating cellular processes includes growth and metabolism, the cell cycle, apoptosis, folding and ER stress ( Figure 1). The mTOR pathway, a key regulatory pathway of energy metabolism, is overexpressed in CHO cells and significantly improves VCD and RTP production by regulating TFs [ 38, 39]. Certain TFs regulate the expressions of several related genes and improve cell proliferation and metabolism. For example, Myc simultaneously modulates genes governing metabolism and the cell cycle. Other TFs are activated when they bind to small molecules, tailoring their response in a specific way depending on the ligand, turning the effect on (or off) when needed, and affecting apoptosis. Such ligand-responsive TFs allow the development of precise control and more predictable biological processes, thus accelerating the development of biopharmaceuticals [29].

### Classification and function of translational factors

**Figure 2:**
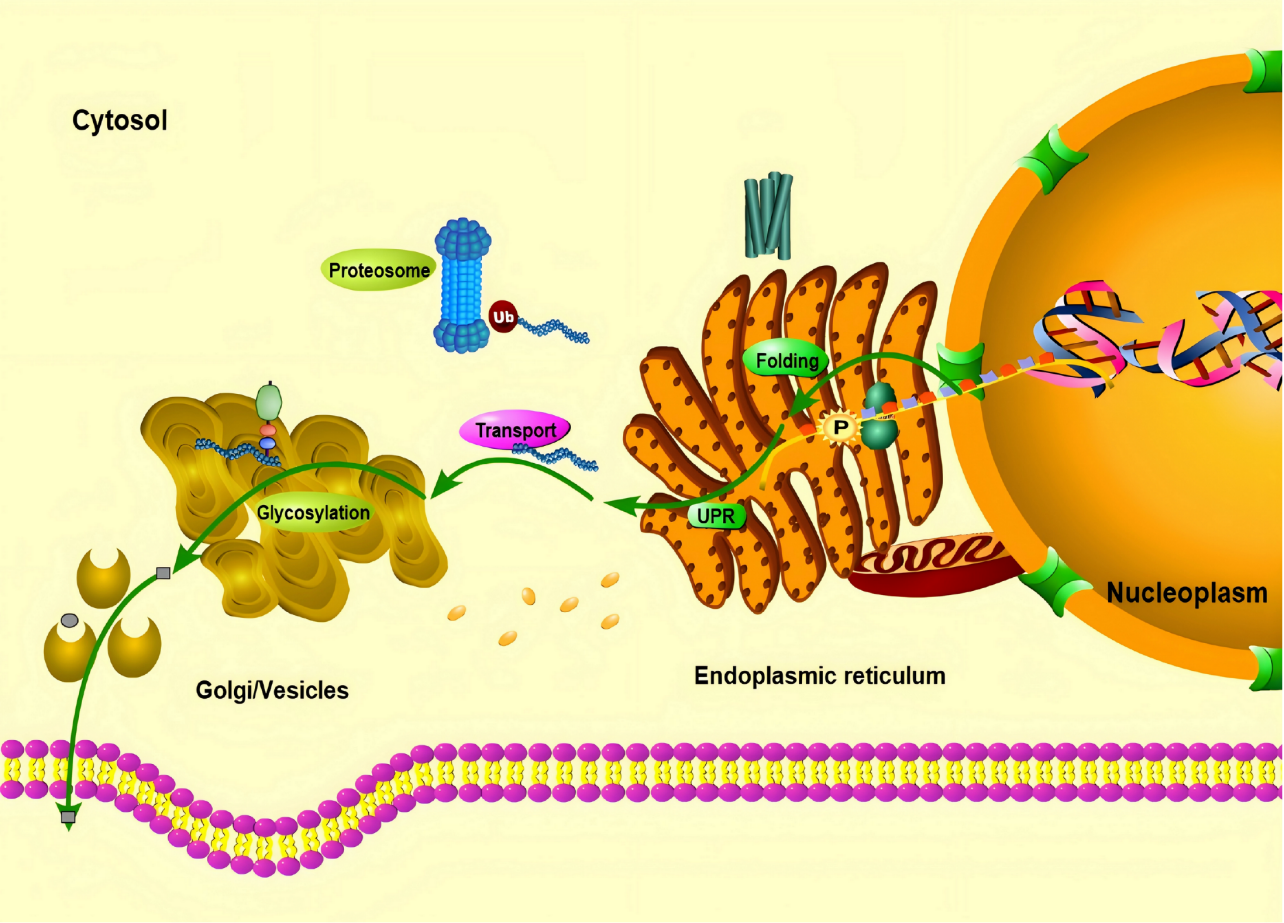
Figure 2 Mechanisms of translation regulators in CHO cells Phosphorylation induces the initiation of translation (two deep blue spheres on the ER: ribosome) and regulates the rates of protein synthesis (curved blue-gray dotted structure), ensuring the correct folding of proteins. The unfolded proteins can be degraded by the proteasome (dumbbell-shaped blue/dark blue spheres; “Ub”: ubiquitin chain), activating the UPR to improve the activity of the ER (brown sac-like/tubular structures with dark dots). Under the assistance of transporters (bright yellow ellipses with internal rings), the correctly folded protein enters the Golgi apparatus (pink ellipse), completing glycosylations (blue/pink/light green circles) and other modifications, and then is secreted into the cell supernatant via vesicles. Cellular context annotations include the following: the plasma membrane (parallel pink spheres with yellow interlayers), the nucleus (orange semicircles), and the nuclear pore complex (green structure on the nuclear membrane).

Translation factors, which include mainly initiation/elongation factors, transcription proteins, non-coding RNAs, and chaperones, are involved in translation, folding, protein transport, secretion and PTMs ( Figure 2). The mTOR signaling pathway plays an irreplaceable role in the regulation of ribosomal small subunit recognition of mRNAs, phosphorylating downstream 4EBPs to promote eIF4F recognition and m7G to promote translation initiation and drive translation elongation by phosphorylating S6 kinase 1 (S6K1). Typically, after recognizing m7G, the ribosome scans from 5′ to 3′, and when the initiation codon is encountered, it recruits eIF2 and GTP and initiates Met-tRNAMet to form a ternary complex and initiate polypeptide chain synthesis. Structural features of the 5′-UTR can significantly affect mRNA translation efficiency, such as the addition of the putative m ^6^A motif to the 5′-UTR when the human glycoprotein erythropoietin (EPO) is expressed. In batch culture, the EPO titer significantly increased with the addition of the m ^6^A structure [40]. The unfolded protein response (UPR) modulates ER activity through three proximal UPR sensors identified in mammals: activating TF6 alpha (ATF6), inositol-requiring enzyme 1 (IRE1), and PKR-like ER kinase (PERK). PERK16 phosphorylates eIF2α, temporarily decreasing protein synthesis and preventing the accumulation of unfolded and misfolded proteins in the ER, subsequently promoting secretion [41]. Effectively regulating the accumulation of fold-related proteins in the ER is an effective way to construct high-yield CHO cell lines. Sar1A overexpression promotes the transport of proteins from the ER to the Golgi apparatus via COPII vesicles, and the specific antibody production rate increases in fed-batch culture [42] .

PTMs in CHO cells can further alter protein function by increasing the interaction of RTPs with other complex factors. The upregulation of PTMs can increase secretory capacity and glycosylation. For example, the secretion efficiency of the BMP protein is closely related to the synthesis of N-glycans. However, limited translational regulation may lead to a bottleneck in CHO cell secretion [43]. In addition, translation regulators can also be non-coding RNAs, particularly microRNAs (miRNAs). miRNAs are short regulatory non-coding RNAs that can regulate cell behavior and complex phenotypes, including promoting proliferation and anti-apoptotic effects and regulating metabolism to improve the yield and quality of RTPs in CHO cells [44].

TFs and translational factors exhibit functional synergy. The improvement of the cell productivity phenotype by TFs is holistic, promoting transcription and enhancing the translation, folding, and PTM processes [45]. In CHO cells with variable genomes, disturbances in the chromatin and nuclear proteomes profoundly affect the productivity of derived clones. PTM-mediated changes in translational factors promote the nuclear translocation of TFs and alter DNA binding and transcriptional reprogramming [46] .

## Effects and Mechanisms of Transcriptional and Translational Regulators on Recombinant Protein Expression in CHO Cells

### Improving recombinant protein expression levels

Improving the productivity of CHO cells requires a better understanding of the cell’s limitations and a potential redesign of protein synthesis and the involved mechanisms. Studies have shown that low expression in CHO cells is associated with a limited ability for transgene transcription, protein synthesis, and processing and secretion of glycoproteins (which we call a “productivity phenotype”), where the transcription process is the rate-limiting step [29]. The simultaneous promotion of cell growth and the production of RTPs is a desirable goal for CHO cell engineering, but such results are difficult to achieve through genetic manipulation. Gene expression driven by TFs is complex and involves cross-regulation and feedback loops to improve the overall adaptability of CHO cells ( Table 2).

**Table:** **Table 2** Improvement in RTP levels by transcription factors in CHO cells

Type	Cell line	Condition	r-Protein	T (°C)	Outcome and reference
CREB	CHO-DHFR	CREB phosphorylation	IgG	36	Showed ~6-fold increased association with the CMV promoter activity [33]
CREB	CHO-GSR	Treated with forskolin	IgG	37	Increased culture longevity and 1.5-fold in the mAb concentration [47]
VP16-CREB	CHO-K1	Constitutively active CREB	Etanercept	37	CMV promoter activity was increased up to 3.9-fold [48]
FoXa1	CHO-M	Overexpression	Trastuzumab and Infliximab	37	Increased cell density, cell viability, and easy- and difficult-to-express protein yields [38]
c-Myc	CHO-K1	Overexpression	hSEAP-hFc	37	More than 70% increase in maximal cell density [49]
c-Myc	CHO TF70R	Temperature downshifts	rh-tPA	33	Increased 2.2-fold in rh-tPA concentration [50]
c-Myc	CHO-DG44	Mild hypothermia	Anti-TNFα	33 to 31	Increased anti-TNFα production [51]
eIF3c	CHOK1	Overexpression	Luciferase protein	30 to 33	Enhanced c-Myc expression and RTPs synthesis [45]
Myc and XBP1s	CHO-K1	Overexpression	EPO	33 to 37	Enhancing EPO production in CHO cells, especially in cultures supplemented with high glucose [52]
YY1	CHO-K1/ CHO-B13-24	Overexpression	Rituximab	37	Increased the antibody titer up to 6-fold [53]
HIF-1α	CHO-K1/CHO-DG44	Overexpression	SEAP	37	Improved production titers under hypoxia induction [54]
E2F1	CHO-DG44	Overexpression	IgG	–	Increased producing a humanized monoclonal antibody and changed cell cycle [55]
Requiem and Alg-2	CHO-RA	Co-overexpression	IFN-γ	37	Increased RTPs productivity by 1.5-fold compared with targeting just Requiem alone [56]
NF-κB	CHO-S/CHO-K1	Knockout	SEAP	37	Reduced CMV-driven SEAP expression [57]
Prox1	CHO	Overexpression	EGFP	37	Increased transfected cell proliferation and EGFP levels [58]
YAP	CHO-K1	Overexpression	EPO	37	Increased 3-fold in total protein and EPO, and 1.5-fold in specific productivity [10]
PGC-1α	CHO	Overexpression	IgG	37	Increased oxidative metabolism and 5.2-fold in QP relative to the parental line [59]
S6K	CHO-K1	High transcript level of S6K	GM-CSF	30	Three-fold increase in GM-CSF production [17]
ZFP-TF(LK52)	CHO-AKA	Overexpression	mAb-72	37	Achieved a 10-fold increase in mAb-72 production with 60% transduction efficiency [60]
ZFP-TF	CHO-DG44	Overexpression	IgG	36.5	Increases the protein production by up to 500% and the stability maintained over > 30 generations [61]
ZFP-TF	CHO-K1	PiggyBac transposon mediate integration	scFv-Fc	37 to 33	High-titer antibody production at the gram-per-liter level was achieved [62]
ZF-GCN4-AD	CHO-K1	MultiFate architecture of more TFs	mTagBFP2	–	Increased mTagBFP2 expression and generate robust stable states [36]

According to differential nuclear proteomic analysis, the activity of cAMP response element-binding protein (CREB) and its association with CMV promoters are increased in high-producing cell lines [33]. Moreover, CREB activation in recombinant CHO cells upregulates the expression of antiapoptotic B-cell lymphoma-2 (Bcl-2), thereby extending culture longevity, suggesting a positive effect of CREB activation on the survival of host cells [47]. In addition, the constructively active TF VP16-CREB has been successfully used as a synthetic promoter to drive the expressions of transgenes containing cAMP-responsive elements. In CMV promoter-driven rCHO cell lines, VP16-CREB increased the production of etanercep by 3.9 folds [48].

The overexpression of FoXa1 increased the cell density, viability, and production of DTE proteins while decreasing the level of reactive oxygen species (ROS) in late fed-batch cultures [38]. c-Myc is a spiral-transspiral-helix TF that regulates the expressions of up to 15% of genes across the entire genome and broadly controls genes associated with cell growth, metabolism, the cell cycle, proliferation, and apoptosis. c-Myc overexpression increased the rate of cell growth by 3.8-fold and reduced the rate of glucose consumption by 3.6-fold compared with those of the controls in CHO-K1 cells. Under mild hypothermia (33°C), the levels of NDK-b (a transcriptional c-myc activator) are elevated, and Qp of EPO is increased in CHO cells [49]. In addition, mild hypothermia upregulates Myc at 30°C, resulting in an increase in hr-tPA single-cell production but a 19% decrease in the lactate/glucose ratio compared with that under 37°C culture conditions [50]. Consistently, elevated c-Myc increased maximum VCD and anti-TNF antibody production in CHO cells [51]. Myc can also be regulated by the mTOR signaling pathway, ribosome biogenesis and the eukaryotic initiation factor eIF3c to increase protein synthesis rates [45]. In CHO cells, YAP5SA overexpression upregulated the c-Myc and mTOR pathways, synergistically influencing the cell cycle and reducing apoptosis, thereby increasing RTP production [10]. Therefore, c-Myc has a positive effect on CHO cell proliferation and metabolism. However, compatibility with promoters directly affects transcriptional activity; thus, synthetic enhancers of transcription factor regulatory elements (TFREs) can improve gene transcription and protein expression [4].

XBP1 is a key regulator of protein processing and the UPR in the ER. Upregulating XBP1 leads to ER enlargement, which is essential for high antibody secretion rates in plasma cells. XBP1 has been shown to increase product titer and cell-specific productivity in CHO cells [63]. Many genes require multiple TFs for expression, and there is a strong correlation between the expression of Myc and that of XBP1 in CHO cells. Latorre *et al*. [52] reported that co-expressing Myc and XBP1 could improve the overall culture performance of the EPO-producing CHO cell line by simultaneously increasing VCD and product yield. This enhancement occurs through two distinct mechanisms: Myc overexpression enhances cell growth, whereas the overexpression of XBP1 increases EPO productivity. The combined effect is an increase in both VCD and overall EPO yield.

Yin Yang 1 (YY1) belongs to the polycomb family and interacts with a variety of proto-oncogenes, such as c-Myc, c-Fos, EGFR, c-ErbB2 and Mdm2. The transcriptional activation or inhibition of YY1 depends on the proteins with which it interacts [ 64, 65 ]. Overexpression of CHO-derived YY1 increased the production of RTPs in CHO cells without affecting their proliferation, suggesting a positive effect of YY1 in CHO cells [53]. Hypoxia occurs during batch culture or perfusion cultures with high cell density. HIF-1α controls gene expression under hypoxic conditions, mediating the expression of various genes associated with oxygen tension. Zeh *et al*. [54] created a hypoxia-responsive cellular system in CHO cells on the basis of HRE elements and cellular oxygen-sensitive proteins, which significantly improved protein expression during batch culture and high-density perfusion. However, another study suggested that the HIF-1 signaling pathway plays a significant role in metabolic changes, resulting in the phenotypic instability of a recombinant CHO cell line [66]. Therefore, under certain conditions, TFs may have opposite effects on the expressions of RTPs in CHO cells.

E2F1 interacts with different proteins, such as pRb or ATM serine/threonine kinase (ATM), to regulate the cell cycle or apoptosis. In batch cultures of CHO-DG44 cells, E2F1 overexpression and cell proliferation were observed for one day, with a 20% increase in cell density, thereby increasing the production of expressed mAbs [55]. Transcription factors play a role in inhibiting mitochondria-mediated apoptosis. Under feeding conditions, Requiem and Alg-2 increased the maximum VCD and cumulative IVCD, resulting in an approximately 1.5-fold increase in RTP production [56]. Simultaneous downregulation of NF-κB- and CRE-mediated retrotranscriptional activation reduces the production of CMV-driven transient SEAP by more than 75% [57]. The homeobox TF (Prox1) was transfected into CHO cells, and several stable Prox1-CHO cell lines were screened and established; compared with GFP-CHO and parent CHO cells, Prox1-CHO cells presented increased EGFP expression [58]. YAP is a transcriptional coactivator that contains five HXRXXS motifs, which are the phosphorylation sites of the LATS kinase. The above five motifs (YAP5SA) were mutated and cotransfected with *EPO* in CHO cells, resulting in a significant 3-fold increase in EPO production and a 1.5-fold increase in specific productivity [10]. Compared with low-yielding cell lines, high-yielding CHO cell cultures presented increased mitochondrial metabolism and overexpression of transcriptional coactivator-1α (PGC-1α), increased oxygen consumption and mitochondrial metabolism and increased mAb-specific productivity [59].

mTOR signaling can simultaneously improve key processes of RTP production in CHO cells, including cell growth, proliferation, viability and cell-specific productivity. mTORC1 directly phosphorylates 4E-BP1, promoting its dissociation from eIF4E, allowing the formation of complexes with eIF4F and the initiation of cap-dependent translation. Increased phosphorylation of 4E-BP1 is associated with increased interferon-γ production in CHO cells due to the reduced inhibition of translation initiation by mTORC1 [39]. The amount of 4E-BP1 in cells may vary across RTP-producing cell lines, and the protein ratio of 4E-BP1 to eIF4E, a central parameter of cap-dependent translation, is associated with a greater degree of cell productivity [67]. S6Ks (S6K1 and S6K2) have been implicated in promoting ribosome biogenesis. Phosphorylation of S6Ks may increase translation through a number of possible mechanisms, including inactivation of kinases that phosphorylate the eukaryotic elongation factor eEF2. Shahabi *et al*. [17] reported that knocking down the upstream suppressor genes *TSC1* and *MAPKAPK5* of mTORC1 increased the transcription level of S6K and cell growth-related properties. Translation efficiency and protein expression are enhanced when the 30°C mild temperature culture process and the use of HSP90 promoters are combined, resulting in a 3-fold increase in the production of granulocyte-macrophage colony-stimulating factor (GM-CSF) protein.

Zinc finger protein transcription factor (ZFP-TF) can rapidly and steadily increase protein production in mammalian cells. Kwon *et al*. [60], using a random library of these TFs, identified four potential ZFPs from a screening pool of 2000 candidate genes that increased antibody expression in CHO cells by 10-fold compared with that in control cells. Under instantaneous, stable and perfusion culture conditions, the protein production of ZFP-TF from CHO cells doubled. The design of expression vectors carrying up to 10 ZFP binding sites further increased ZFP-mediated protein production by 5-fold [61]. Ying *et al*. [62] used a ZFP-TF to construct a tetracycline-based transcription activation system in CHO cells and integrated multiple copies of scFv-Fc into the cell genome via a TRE-driven promoter. By combining low-temperature culture conditions, high-density semi-continuous culture was achieved, which can increase the concentration of the scFv-Fc antibody produced [62]. Fusing the ErbB2 ZF DNA-binding domain with the GCN4 homologous dimeric domain and the VP48 transcriptional activation domain created a synthetic TF, ZF-GCN4-AD. A transcription factor lacking GCN4 (ZF-AD) was used as a monomer control to co-transfect CHO-K1 cells with a reporter gene. Flow cytometry revealed that ZF-GCN4-AD strongly activated reporter gene expression [36]. The increased demand for more DTE proteins or low-expression proteins requires further exploration of the effects of ZFP-TF on a given promoter, enabling the synthesis of stronger promoters.

The regulation of protein translation involves complex mechanisms, including epigenetic modifications and miRNAs ( Table 3 ). m ^6^A is the most abundant modification in eukaryotic mRNAs and is involved in all aspects of RNA metabolism, including splicing, stability, translation, and microRNA processing. siRNA-mediated gene deletion of m ^6^A YTH domain-containing readers (YTHDF2) increased the levels of GFP and EPO recombinant proteins in CHO cells (~2-fold) [35]. Transcriptome sequencing revealed that in CHO cells overexpressing YTHDF3, multiple genes involved in translation initiation, extension and ribosome assembly factors were upregulated. Stable expression of YTHDF3 significantly enhanced the expression of RTPs in CHO cells without affecting the growth of host cells. Cycloheximide experiments confirmed that YTHDF3 enhances the translation of transgenes in CHO cells [68]. Therefore, epigenetic factors play important roles in RTP translation, necessitating further screening of key epigenetic factors. Codon preference can regulate the elongation of the translation process to improve translation efficiency. The heterologous expression of the recombinant human interferon ( *rhIFN-β*) gene in CHO-S cells increased the GC content at the third codon position, increasing expression by 2.8-fold [69]. *Escherichia coli* produces the non-glycosylated form of IFN-β 1b, while the more bioactive glycosylated form of IFN-β 1a is produced by CHO cells. Oligosaccharides play crucial roles in the stability, solubility, and immunogenicity of rhIFN-β, inhibiting *in vitro* aggregation and enhancing *in vivo* biological activity [70]. Small molecule inhibitors targeting CDK phosphorylation can manipulate cell cycle G1 checkpoints in CHO cells, thereby controlling cell proliferation and increasing specific productivity [71]. Moreover, ectopic mTOR expression is correlated with improved ribosome biosynthesis in CHO cells, where mTOR-transgenic cell lines achieved 4-fold greater antibody titers [39]. The deletion of pyruvate dehydrogenase kinase PDHKs and lactate dehydrogenase reduces the phosphorylation of pyruvate dehydrogenase, increasing the specific yield and reducing lactic acid production without significantly affecting cell growth [72].

**Table:** **Table 3** Improvement in RTP levels by translation regulators in CHO cells

Type	Cell line	Condition	r-Protein	T (°C)	Outcome and reference
YTHDF2	CHO-K1	Knockdown by siRNA	EPO/EGFP	37	Enhanced the levels (~2-fold) of GFP and EPO transgenes in CHO cells [35]
YTHDF3	CHO-S	Overexpression	Adalimumab	37	YTHDF3 enhanced transgene expression by promoting translation [68]
CDK4/6	CHO	Selectively inhibitor	IgG	37	Specific productivities increased up to 110 pg/cell/day [71]
mTOR	CHO-K1	Overexpression	IgG	37	Achieved four-fold antibody titer increase compared to control cell line [39]
LDHa/PDHKs	CHO-DUXB11/DHFR	Knockdown by siRNA	IgG	37	Increased specific productivity and volumetric of antibody production [72]
miR-574-3p	CHO-DUXB11	Overexpression	EPO and ETN	37	Improve productivity of EPO and Etanercept titers from 1.3 to 2 folds [74]
miRNA-7	CHO-DP12	Sponge depletion	IgG	37	Increased cell growth by 65% and 3-fold increase in yield of secreted IgG protein [75]

miRNA-targeting cell engineering strategies may alter the entire transcriptional pathway without disrupting cellular translation mechanisms. miRNAs are non-coding, enabling the cellular processing machinery to prioritize protein production [73]. Stable overexpression of miR-574-3p in CHO-DHFR fine cells promoted transcriptional capacity and increased target gene mRNA abundance, and RTP production increased by an average of 30%–40% after 4 days of culture. The increase was even more pronounced in larger batch cultures, with EPO expression increasing 2-fold compared with that in control cells [74]. However, the inhibition of miRNA-7 in CHO-DP12 cells impaired ribosome biogenesis but improved ribosomal translation activity, resulting in a 15-fold increase in RTP expression compared with that in controls [75]. Therefore, miRNAs have dual effects on protein expression, which are primarily determined by target genes.

#### Promotion of protein folding and secretion

Limited posttranslational processing and retention in the Golgi apparatus lead to secretion bottlenecks in CHO cells [76]. Developmental reprogramming of B cells is synergistically regulated by three transcription factors: interferon regulatory factor 4 (IRF4), B lymphocyte-induced maturation protein (Blimp1) and XBP1 ( Table 4). IRF4 is essential for plasma cell development and survival by activating and maintaining high expression of Blimp1 in differentiated plasma cells. High IRF4 expression increases mitochondrial mass and controls the production of ROS during differentiation, which is linked to metabolic regulation. Blimp1, the master transcription factor of plasma cells, is highly expressed in all antibody-secreting cells; ectopic expression induces plasma cell differentiation in mature B cells [77]. Overexpression of Blimp1 decreased the cell density and significantly increased the ER size and protein synthesis and secretion capacity. Recombinant IgG1 resulted in significantly higher titers (> 2-fold increase) and cell-specific yields (> 3-fold increase) and improved cell production phenotypes [78]. In particular, the expression of Blimp1β induces different gene expression patterns, thus promoting protein processing in secretory organelles. In CHO cells that produce DTE recombinant human bone morphogenetic protein-4 (rhBMP-4), the secretion of mature rhBMP-4 is promoted, significantly increasing the rhBMP-4 concentration (> 3-fold) and specific rhBMP-4 productivity (> 4-fold) without affecting hBMP-4 transcription levels [79]. Similarly, Blimp1 expression enhances target protein secretion in CHO cells producing IgG1 and EPO-Fc fusion proteins [78]. Consistent with these results, the combination of Blimp1 and NaBu treatment led to an additive effect on cell cycle arrest in the G1/G0 phase and maximally improved the titer and QP of RTPs and increased the product yield (IgG1 up to 9.8 g/L and EPO-Fc 2.2 g/L) and QP up to 179 pg/cell/day (IgG1) and EPO-Fc up to 30 pg/cell/day in high-density perfusion cultures [80].

**Table:** **Table 4** Promoting protein folding and secretion in CHO cells

Type	Cell line	Condition	r-Protein	T (°C)	Outcome and reference
Blimp1	CHO-K1 /CHO-S	Overexpression	EPO/IgG1	37	Increased product titer of recombinant IgG1 (> 2-fold) coupled with cell-specific productivities (> 3-fold) [78]
Blimp1β	CHO-DG44	Overexpression	rhBMP-4	37	Increased the rhBMP-4 concentration (> 3-fold) and specific rhBMP-4 productivity (> 4-fold) [79]
Blimp1	CHO	Overexpression and NaBu	EPO/IgG 1	37	Increased product yields (9.8 g/L IgG1 and 2.2 g/L EPO-Fc) and QP (179 pg/cell/day and 30 pcd for EPO-Fc) in high-density perfusion cultures [80]
XBP1	CHO-K1	Co-overexpression	IgG1/IFNc/EPO	37	Overcome secretory bottleneck and improved EPO titers by up to 2.5-fold [81]
Xbp1/ERO1-La	CHO-S	Co-overexpression	IgG	37	Increased mAb yields (5.3–6.2-fold) in comparison to CHO-S cells [82]
Blimp1/XBP1	CHO-K1 /CHO-S/CHOB	Co-overexpression	IgG1/hEPO-Fc	37	Increase cell-specific productivities (9-fold) and product yields (3-fold) for different r-proteins [83]
Ero1/PDI	CHO	Transient coexpression	IgG1	37	Promoted mAb folding and secretion and enhanced Qp by 55% [84]
PDIa4	CHO-HcD6	Knockout	IgG1	37	Decreased the amount of secreted antibody and the accumulation of immature mAb inside the cells [31]
PDI/ XBP-1s	CHO-S	Co-overexpression	Adalimumab	33	Adalimumab volume yield increased by 203% [24]
Erp27/Erp57	CHO-M	Co-overexpression	infliximab	37	Resulted in a 72% increase in infliximab titers [38]
QSOX1b/survivin	CHO-K1	Knock in	GLuc	37	Improved ER microenvironment and the yield of Gluc was raised 5.55-fold [85]
QSOX1b/survivin	CHO	Co-overexpression	IgG1	37	Accelerated disulfide bond folding capacity; antibody accumulation and productivity were increased by 52% and 45% [21]
BiP, ATF6c and XBP1s	CHO-K1	Co-overexpression	IgG	36.5	Improved Qp of DTE mAbs and the rate/capacity of folding and assembly reaction [86]
ATF6β	CHOK1SV GS-KO	Knockdown and fed-batch	IgG1	37	Increased cell specific daily (day 9 and 11) and overall productivity [87]
ATF4	CHO-K1	Overexpression	AT-III	37	Enhanced the production of recombinant AT-IIIin CHO 13D-35D cells [88]
NFKBIZ	CHO	Overexpression	IgG1	37	The specific IgG1 production rate of both cell lines was enhanced by 1.2 to 1.4-fold [89]
ATF4/SRP9	ExpiCHO-S	Co-overexpression	THBS4	37	More than two-fold improvements in secreted THBS4 titers [90]
ATF4/JUN	ExpiCHO-S	Co-overexpression	THBS4	37	A 1.5-fold titer increase [90]
GADD34	CHO 13D-35D	Overexpression	AT-III	37	The specific rate of AT-III production reached approximately 28 pg/cell/day, approximately 40% higher than control [91]
SREBF1/SCD1	CHOK1SV GS-KO	Co-overexpression	IgG/Fc fusion protein	37	Expression of secretory RTPs was enhanced between 1.5- to 9-fold in either SREBF1 or SCD1 engineered CHO host [92]
CERT	CHO-DG44	Overexpression	HSA/IgG	37	Enhanced mAb secretion and overall HSA titers [93]
HSP27	M250-9	Overexpression	IgG	37	In fed-batch bioreactors displayed 2.3-fold increase in mAb titer, without affecting the N-glycosylation profile [94]

The ER is critical for the correct synthesis and assembly of recombinant secreted proteins, and the levels of secretion required for industrial CHO cell lines can challenge ER homeostasis, whereas ER stress from the accumulation of misfolded proteins creates a bioproduction bottleneck [87]. During ER stress, the UPR is activated to restore homeostasis, and UPR optimization can improve the production of RTPs in CHO cells. XBP1 overexpression during transient expression increases the expression of mAbs, IFNc, and EPO [81]. An engineered CHO-XE cell line was developed by Cain *et al*. [82] on the basis of the overexpression of XBP1s and ERO1-Lα, which is necessary for disulfide bond formation. This alteration of the ER environment enhances both VCD and cell viability, resulting in a 5.3–6.2-fold increase in antibody production [82]. High RTP expression intensifies the burden of secretion in the ER, resulting in a secretion bottleneck and decreased production. XBP1s improves secretory capacity by promoting multi-gene expression [95]. Cell engineering strategies based on double Blimp1 and XBP1 overexpression effectively reprogrammed CHO cell secretion mechanisms. Blimp1 overexpression arrested the cell cycle in the G1/G0 phase and increased CHO cell protein synthesis, whereas XBP1 overexpression expanded the ER and improved its capacity for protein secretion, enabling CHO cells to gain additional secretion capacity. In addition, the co-expression of Blimp1 and XBP1s altered the cellular metabolic pattern, characterized by a pronounced lactate switch and increased amino acid consumption. These studies suggest that XBP1-mediated RTP enhancement in CHO cells requires cooperation with other factors capable of increasing cell biosynthesis [83]. Therefore, the regulation of ER folding and secretion in CHO cells requires the simultaneous participation of multiple factors, with the co-expression of TFs and translation factors being particularly effective for improving RTP processing.

PDI and ER oxidoreductase 1 (Ero1) catalyze disulfide bond formation in newly folded proteins. Mohan *et al*. [84] reported that transient co-expression of Ero1 and PDI in antibody-producing CHO cells increased specific antibody production by 37% and 55%, respectively. When *PDIa4* was knocked out in CHO cells, a decrease in antibody secretion and the accumulation of immature antibodies in the cells were observed. Moreover, the addition of recombinant PDIa4 can refold the antibody [31]. Compared with those of the control/37°C and PDI/XBP-1s/37°C groups, adalimumab volume production was increased by 203% and 142% for the PDI/XBP-1s/33°C group, respectively. Mild hypothermia resulted in 3.52-fold and 2.33-fold increases in the relative mRNA levels of PDI and XBP-1s, respectively [24]. Erp27, a PDI family member, binds to unfolded protein and Erp57 disulfide isomerase in the ER, as does Foxa1, a precursor TF involved in organ development. Moderate Erp27 overexpression increased the production of antibodies, whereas co-overexpression of Erp27 and Erp57 increased cell density, viability, and DTE protein yields [38]. Sulfhydryl oxidase 1 (QSOX1) is involved in disulfide bond formation and transfer, and its enzyme activity is 100-fold greater than that of Ero1 sulfhydryl oxidase. Survivin has been shown to inhibit cysteinyl aspartate-specific proteinase-3/7 activity, thereby directly inhibiting apoptosis. HsQSOX1b and survivin co-overexpression in CHO-K1 cells can increase disulfide bond formation and the folding ability of humanized Gaussia luciferase (GLuc), improve anti-apoptotic ability, and prolong the cell protein production cycle [85]. Consistent with this, HsQSOX1b and survivin co-overexpression increased the UPR intensity and increased the ER content, promoting protein disulfide bond folding and the anti-apoptosis ability of CHO series of cell lines and improving antibody productivity [21].

ATF6 is one of the three typical UPR sensors, and co-expression of ATF6α and XBP1 decreases the cell growth rate (dependent upon the expression level) regardless of the specific mAb used, leading to a reduction in IVCD but not in cell viability. Furthermore, ATF6α and XBP1 enhance RTP folding and assembly, improving the UPR in transient gene expression systems and ultimately increasing antibody production while decreasing cell proliferation [86]. However, ATF6β negatively regulates ATF6α, and its knockout amplifies the UPR, thus improving overall productivity [87]. ATF4 can respond to hypoxia, ER stress, amino acid deprivation and oxidative stress and significantly improves the production and secretion of recombinant human antithrombin III (AT-III) in CHO cells [88]. Stable expression of the NF-κB inhibitor zeta in cell lines improved the synergistic effect between VCD and specific IgG1 production and enhanced the secretion of correctly folded IgG1, increasing the final titer by 1.2–1.4-fold [89]. In CHO cells, the expression of RTPs was significantly increased when cotransfected with the *ATF4*, *SRP9* and *JUN* genes, which are involved in protein secretion. ATF4 or SRP9 co-expression doubles the transgenic titer secreted in CHO cells, and when co-expressing with JUN, the titer was increased by 1.5 folds [90]. The transcription factor GADD34 mediates the UPR in CHO-k1 cells by regulating growth arrest and DNA damage induction. Overexpression of GADD34 significantly enhanced the production of recombinant AT-III in CHO 13D-35D cells [91]. Global transcriptional activators of genes involved in lipid biosynthesis and co-overexpression of sterol regulatory element binding factor 1 (SREBF1) and stearoyl-CoA desaturase 1 (SCD1) amplified RTP secretion by 1.5–9-fold in batch/fed-batch cultures, resulting in ER expansion [92].

Ceramide transfer protein (CERT) mediates the transport of ceramide from the ER to the Golgi apparatus. Florin *et al*. [93] reported that ectopic expression of CERT and its mutant CERT S132A enhanced the secretion of mAbs and HSA in recombinant CHO cells. Heat shock proteins (HSPs), which are located primarily in the cytoplasm, prevent protein aggregation and participate in the refolding of misfolded proteins. Compared with control clones, stably overexpressing HSP27 in CHO cells produces recombinant monoclonal antibodies, and clonal live cells cultured in batch bioreactors stably transfected with HSP27 have a 2.2-fold greater VCD and 2.3-fold greater mAb titer without affecting the N-glycoylation profile [94]. Additionally, PDIA3 and HSPA8 significantly improved the secretion of DTE proteins. Co-overexpression of HSPA1B and SRP9 elevated the titer of ARTN. Compared with those of the controls, higher secreted ARTN titers in CHO cells were associated with lower VCD and cell viability at harvest, thereby increasing RTP production [90].

#### Improving protein quality

The quality properties of recombinant proteins, including charge variants, aggregation, degradation, and glycosylation, directly affect their safety, activity, pharmacokinetics and pharmacodynamics [14]. In CHO cells, PTMs include mainly glycosylation, phosphorylation and ubiquitination and are crucial for mediating protein conformation, localization and stability ( Table 5). Glycosylation significantly influences protein stability and folding, the immune response, biological activity, and the serum half-life; thus, it is considered the key quality attribute for RTPs [96]. Identifying new methods for reducing fucose in mAbs has important implications for the biopharmaceutical industry. Wang *et al*. [97] applied CRISPR/Cas9 to knockout the *FUT8* gene, eliminating α-1,6 fucosylation on antibodies expressed in CHO cells. The fucosylation regulatory factor genes *FUT8* and *SLC35C1* were knocked out via gene editing technology. Both the expressed EPO-Fc and anti-Her2 antibodies lacked core fucosylation, and these mutant cell lines did not affect cell growth or antibody production [98]. In addition, another study revealed that mAbs produced by control cells had normal fucose (mainly G0F glycan), whereas mAbs produced by *Fut8*- and *Slc35c1*-KO double-knockout cells lacked fucosylation (mainly G0 glycan) [14]. Fischer *et al*. [99] obtained a single point mutation in the promoter region of cell adhesion molecule 1 (SDK1) that caused the loss of cgr-miR-111. Silencing of *SDK1* and *miR-111* inactivates (CMP)-N-acetylneuraminidase hydroxylase (CMAH) expression, increasing the sialylation of N-glycosylneuraminidase (NGNA) in recombinant mAbs. Moreover, overexpression of N-acetyllactosaminide alpha-1,3-galactosyltransferase (GGTA) and CMAH in CHO cells resulted in the production of glycans with galactose-α-1,3-galactose (alpha gal) and N-glycolylneuraminic acid (Neu5Gc) [100]. Li *et al* . [101] developed a novel CHO-K1-cell line in which the *C1s* and *MGAT1* genes are inactivated by gene editing, restricting glycosylation to mannose-5 or earlier intermediates in the N-linked glycosylation pathways. In CHO-K1 cells, overexpression of miR-106b can directly target the deubiquitinated 3′UTR and reduce CYLD mRNA and protein levels, thereby inhibiting degradation and enhancing antibody expression [102]. The results revealed that mmu-miR-193b-3p and mmu-miR-452-5p could increase the galactosylation of recombinant proteins. However, mmu-miR-7646-5p, mmu-let-7c-1-3p, mmu-miR-1668, mmu-miR-7243-3p, mmu-miR-6403, and mmu-miR-7665-3p reduce the degree of galactose [103]. In suspended CHO-K1 cells, miR-3096b-5p is a strong regulator, and the artificial design of miR-3096b-5p induces the transformation of cell metabolism and completely eliminates the fucose phenotype through fucokinase (FUK) downregulation [104]. Engineering host expression systems modulates mAb patterns associated with salivation. In CHO cells, overexpression of α-2,6-sialyltransferase increases α-2,6 sialylation, and gene editing is used to disrupt the enzymes responsible for 2,3-sialylation, thereby reducing 2,3-sialylation levels and increasing ADCC activity [105] .

**Table:** **Table 5** Improvement in the quality properties of RTPs in CHO cells

Type	Cell line	Condition	r-Protein	T (°C)	Outcome and reference
SLC35C1	CHO-K1	Knockout	EPO-Fc/anti-Her2	37	Led to the production of glycoproteins with fucose-free N-glycans [98]
Fut8/Slc35c1	CHOZN	Knockout	IgG1	37	Both Fut8- and Slc35c1-KO cells produced mAbs that are lacked fucosylation (predominantly G0 glycans) [14]
SDK1/miR-111	CHO-DG44	A single point mutation	IgG4	37	Upregulation of CMAH expression and led to upregulation of Neu5Gc acid sialylation on mAbs [99]
miR-106b	CHO-K1	Overexpression	IgG	37	Achieved 0.66-fold titer promotion of IgG by inhibiting deubiquitinase cylindromatosis-mediate degradation [102]
miRNAs	CHO-K1	Overexpression	IgG1	37	miR-452-5p and miR-193b-3p overexpression increased galactosylation, while galactosylation decreased by miR-7646-5p, miR-7243-3p, miR-7665-3p, miR-1668, let-7c-1-3p, and miR-6403 [103]
miR-3096b-5p	CHO-K1	Artificial design/Overexpression	IgG1	37	Induced cellular metabolism shift and completely eliminating the afucosylation phenotype [104]
ST6Gal1	CHO-K1	Overexpression	EPO	–	Generated an IgG with increased levels α-2,6 sialylation [105]
miR-30	CHO	Overexpression	SEAP	37	Triggered protein ubiquitination and increased cell-specific productivity of SEAP [106]
miR-378-3p	CHO DP12	Depletion	IgG1	37	Upregulated ubiquitin carboxyl-terminal hydrolase 14 and RTPs degradation [107]

Phosphorylation is regulated by protein kinases, including serine threonine kinases (STKs) and tyrosine kinases (TKs), whose functions involve modifying proteins by adding phosphate groups. A holistic phosphorylated proteomics study examined the molecular basis of the high/low-Qp phenotype of industry-associated CHO cell lines. Previous studies have shown that amino acid-related protein phosphorylation is increased in high-Qp cell lines and that cell cycle-related protein expression is increased in low-Qp cell lines [108]. Sodium butyrate is a known histone deacetylase inhibitor that improves productivity by increasing the transcriptional mechanism of transgenes. Thus, productivity can be increased by altering DNA-protein interactions in cells [109].

Protein ubiquitination is a common modification associated with degradation and fragmentation that directly affects protein quality. Fischer *et al*. [106] reported that the miR-30 family mediates the ubiquitin pathway by targeting the ubiquitin E3 ligase S-phase kinase isolate protein 2 (Skp2) in CHO cells. During CHO cell culture in which recombinant HSA-HGF (R494E) protein was expressed, intracellular degradation increased on day 8 because ubiquitin ligase activity increased on day 4. The addition of the ubiquitin ligase inhibitor thalidomide inhibited degradation and increased HSA-HGF expression [110]. The regulator of ubiquitin ligases, cullin-associated NEDD8-dissociated protein 1 (Cand1), participates in the degradation of unfolded proteins and affects the degree of fragmentation of mAbs in CHO cells. Cand1 expression was positively correlated with specific productivity [111]. Costello *et al*. [107] demonstrated that the role of ubiquitin carboxy-terminal hydrolase 14 (USP14) can promote CHO cell growth. Another study revealed that the ubiquitin proteasome system plays a role in the degradation of antibodies in CHO cell lines by mediating ER-related degradation [112]. During CHO cell fed-batch culture, low temperature alters the expression pattern of ER degradation and enhances α-mannosidase-like protein 3 and E3 ubiquitin protein ligase synoviase proteinase 1, modulating the UPR/ERAD pathway. They mediate the processing, folding and degradation of proteins in the ER, thereby improving the production and quality of the EPO protein [23]. Proteomic studies revealed downregulated genes related to proteolysis, including E3 ubiquitin protein ligase and ubiquitin, from the 6th to the 8th day of fed-batch culture, confirming that feeding can increase protein synthesis and inhibit protein hydrolysis in CHO cells [113]. Protein ubiquitination or degradation plays a paradoxical role in the ER for misfolded protein degradation, helping to reduce the ER burden and improve RTP production [3]. However, degradation at other stages of production reduces the quality and inhibits the function of RTPs, thus inducing immunogenicity. Therefore, strategies to inhibit degradation are needed to improve and maintain the quality of RTPs in CHO cells.

**Figure 3:**
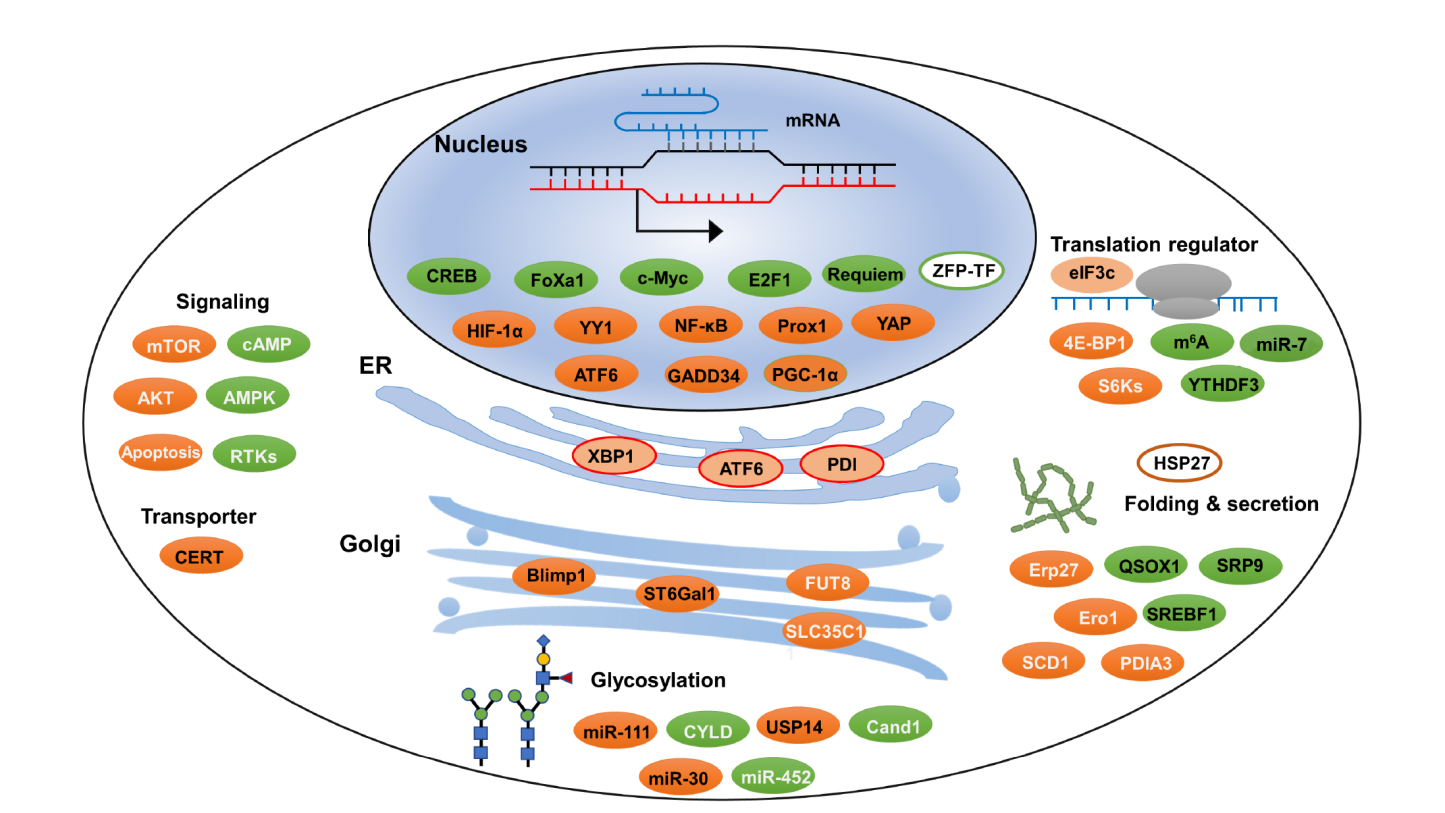
Figure 3 Schematic of transcriptional and translational regulators that increase RTP production in CHO cells Nuclear compartment: Transcription factors (dark green, orange, and white ellipsoids with black labels) that increase RTP expression are labelled in the nucleus, and translational regulatory factors that affect protein folding, secretion (green/orange ellipses with black/white labels), and PTMs are listed in the ER and Golgi apparatus (red box regions with orange backgrounds; orange ellipses with black/white labels).

Currently, most CHO cell engineering studies rely on the overexpression of exogenous TFs and translation regulators, covering several key areas, such as gene transcription, protein synthesis, folding, secretion, and glycosylation, profoundly increasing the yield and quality of RTPs ( Figure 3). However, few studies have investigated endogenous regulatory factors in CHO cells. Thus, future studies should prioritize screening genes with conservative functions in CHO cells to gain an in-depth understanding of the functions of regulatory factors, changing the use trend of endogenous CHO proteins and increasing RTP expression.

## Clinical Efficacy and Implementation Challenges

CHO cells are advantageous for RTP production because of their complete PTM ability and complex protein folding and assembly abilities, making them promising for clinical applications. For example, expressing the spike protein in CHO cells yields high-titer virus-like particles (VLPs) that elicit immune responses at least 3-fold stronger than those induced by the natural virus. Preclinical studies in rodent models have demonstrated that purified SARS-CoV-2 VLPs exhibit remarkably strong efficacy as vaccine antigens, providing effective protection even at submicron doses when combined with commercial adjuvants [114]. Similarly, fusion of the human α1(I) collagen C-terminal peptide (Trimer-Tag) to the C-terminus of mature human TNF-related apoptosis-inducing ligand (TRAIL) results in high-level secretion of the fusion protein from CHO cells. This Trimer-Tag-TRAIL retains *in vitro* biological activity and receptor binding kinetics comparable to those of natural TRAIL while exhibiting improved *in vivo* pharmacokinetic and antitumor pharmacodynamic characteristics [115]. However, a significant challenge with recombinant antibodies derived from CHO cells is their generally high fucosylation, which can negatively impact ADCC. By the use of genetic engineering techniques to reduce fucosylation, folding accuracy, pharmacokinetic properties, and clinical efficacy can be enhanced; for example, defucosylated antibodies exhibit up to 100-fold greater ADCC *in vitro* than fucosylated antibodies from wild-type CHO cells [97]. Consequently, RTPs produced by CHO cells have demonstrated encouraging outcomes, and further modification and optimization of CHO cells holds promise for achieving even better clinical efficacy.

Challenges remain in both recombinant protein (RTP) expression in CHO cells and their translation to clinical use. These include optimizing expression technology for specific target proteins, minimizing the degradation of RTPs by endogenous proteases, bridging the gap between laboratory-scale and industrial-scale production, meeting regulatory standards for cell line stability, and managing the economic factors associated with the expression system. Addressing these challenges requires the integrated application of multiple optimization strategies to enhance the yield and quality of RTP production in CHO cells in combination.

## Conclusions and Prospects

CHO cells remain the predominant platform for the industrial production of RTPs. In recent decades, the yield of RTPs has increased significantly, and improvements in cell line engineering by using transcription and translation regulators is a powerful strategy. However, the production cost of CHO cells is still high, and further screening of key regulatory factors is needed.

A promising direction involves single-cell omics techniques to better understand cell specificity, accelerating screening new candidate genes and elucidating new mechanisms to promote RTP production. In the future, the practicality of TF engineering could benefit from designing a unique TF-binding domain and avoiding recognition of the promoter of host cells to avoid unexpected off-target effects that induce cytotoxic effects. In addition, CRISPR/Cas9 technology facilitates highly specific TF and translation factor adaptation hotspot screening and site-specific integration, resulting in more direct and efficient improvement of RTP expression. With the growing demand for complex fusion proteins, customized CHO cell expression systems will become essential. With strong adaptability, customized artificial TFs and rational development of translation factors are expected to become more specific and critical steps in biotechnology, allowing more robust and efficient designs to meet future demands. In summary, transcription and translation regulators hold significant potential for advancing CHO-based manufacturing.
